# Acyclovir induces testicular damage and impairs HPT axis by upregulating oxidative enzymes and inflammatory cytokines in male Wistar rats

**DOI:** 10.5935/1518-0557.20250022

**Published:** 2025

**Authors:** OO Obembe, RA Mustapha, ET George, BJ Dare, TG Atere, RE Akhigbe

**Affiliations:** 1 Department of Physiology, Osun State University, Osogbo, Osun State, Nigeria; 2 Department of Anatomy, Osun State University, Osogbo, Osun State, Nigeria; 3 Department of Biochemistry, Osun State University, Osogbo, Osun State, Nigeria; 4 Department of Physiology, Ladoke Akintola University of Technology, Ogbomoso, Oyo State, Nigeria; 5 Reproductive Biology and Toxicology Research Laboratory, Oasis of Grace Hospital, Osogbo, Osun State, Nigeria

**Keywords:** acyclovir, antiviral, inflammation, oxidative stress, antiandrogenic

## Abstract

**Objective:**

Acyclovir is an antiviral drug that is used to treat herpes virus infections and acts by inhibiting viral DNA synthesis. While antiviral drugs are designed to inhibit viral replication, some have been found to have immunomodulatory effects beyond their direct antiviral action. Acyclovir has been documented to induce cytotoxicity and DNA mutation. Cytotoxic agents are well-documented to damage male gonadal functions. Therefore, it has become imperative to examine the effects of acyclovir on male reproductive physiology.

**Methods:**

Eighteen adult male Wistar rats were randomly grouped into three groups: control (distilled water), low-dose (10 mg/kg acyclovir), and high-dose (40 mg/kg acyclovir). After 21 days of oral treatment, serum, testicular homogenate, and epididymal sperm suspension were collected and analyzed. Serum and testicular oxidative stress markers (SOD, MDA, GPx, and CAT), hypothalamic-pituitary-gonadal hormones (GnRH, LH, FSH, and testosterone), sperm parameters, and testicular histoarchitecture were examined. In addition, inflammatory cytokines (IL-1β, IL-6, IL-10, TNF-α) and lactate dehydrogenase enzymes were evaluated from the serum.

**Results:**

Acyclovir (40 mg/kg) caused a significant increase in serum inflammatory cytokines (TNF-α, IL-6), LDH, and MDA, while the testicular and serum antioxidant enzymes were reduced when compared with controls. Acyclovir (40 mg/kg) also decreased serum GnRH, LH, FSH, and testosterone levels, as well as testicular testosterone, and negatively affected sperm count, sperm motility, and sperm morphology. Histopathological examination showed that acyclovir caused edematous seminiferous tubules with degenerated spermatogenic cells and scanty sperm cells.

**Conclusions:**

Acyclovir induced testicular damage by promoting inflammatory response, oxidative damage, and endocrine disruption.

## INTRODUCTION

Antiviral medications are bioactive compounds that are produced either by biological organisms or synthesized through chemical processes. They act via the inhibition of viral replication, thus disrupting the capacity of viral entities to infiltrate host cells, undergo uncoating, mature, or replicate. A notable antiviral compound that has gained considerable prominence over the past two decades is acyclovir.

Acyclovir is primarily used in the treatment of herpes viruses, such as herpes simplex virus (HSV), Varicella-Zoster virus (VZV), and Epstein-Barr virus. Its mechanism of action involves the inhibition of viral DNA synthesis, thereby inhibiting DNA replication (Kłysik *et al.*, 2020). The therapeutic efficacy of acyclovir depends on two specific viral proteins; thymidine kinase and endogenous deoxyguanosine triphosphate (dGTP). Thymidine kinase facilitates the entry of acyclovir into the virus and phosphorylates it into acyclovir phosphates while the phosphorylated acyclovir then competes with the viral dGTP for incorporation into the viral DNA, thus causing inhibition and the chain termination of the virus. Despite the effectiveness of acyclovir in combating viral infections, prior investigations have indicated that it may exhibit cytotoxic effects. Furthermore, acyclovir possesses mutagenic characteristics attributable to its influence on cellular DNA (Ekmekyapar & Gürbüz, 2019).

In addition to targeting the viral pathogens, antiviral medications may induce adverse effects in the host organism (Ma *et al.*, 2020; Akhigbe & Hamed, 2021; Akhigbe *et al.*, 2021; Hamed *et al.*, 2021). Acyclovir may also induce organ damage via the induction of oxidative stress (Adikwu & Kemelayefa, 2021) and inflammation (Hui *et al.*, 2020). Acyclovir has been reported to induce testicular damage, reduce testosterone levels, sperm count, and motility, and increase sperm abnormalities by enhancing lactate dehydrogenase release and suppressing Leydig cell function (Narayana, 2008). Movahed *et al.* (2013) also revealed that acyclovir induces testicular damage and causes a reduction in testosterone levels. Despite the wide use of acyclovir and its reported adverse effects, there is a dearth of information on the effects and associated mechanism of action of acyclovir on testicular function viz. testosterone production and spermatogenesis. Therefore, this study assessed the impact of acyclovir on sperm quality and testosterone levels. Also, the roles of oxidative stress and cytokine-driven inflammation were explored.

## MATERIALS AND METHODS

### Animal care and grouping

Eighteen adult male Wistar rats (130 - 170 g) were housed in plastic cages with netted covers in the animal housing of the Department of Physiology Animal House, Osun State University, Nigeria. The rats were given two weeks to acclimatize fed with a standard pellet diet and given unrestricted access to clean water. The Guide for the Care and Use of Laboratory Animals (National Research Council (US) Committee for the Update of the Guide for the Care and Use of Laboratory Animals, 2011) were followed in the handling and care of the animals. After acclimatization, the rats were randomly grouped into three groups of six rats each; Group A received distilled water (solvent), Group B received 10 mg/kg b.w/day of acyclovir, while Group C received 40 mg/kg b.w/day of acyclovir. All treatment was administered orally per day as a single dose for 21 days using an oral cannula (18 G). Oral administration was done because it is the commonest route of administration of acyclovir and to minimize the suffering (from injections) in rats.

The sample size was determined by power analysis using G*Power (version 3.1.9.4) and it was ensured that the final sample size followed ethical considerations. An effect size of 0.7 was used as obtained from our pilot study using Cohen’s d calculator, with a power of 80%, and type 1 error of 5%. Afterwards, the obtained sample size was adjusted for a 20% attrition rate.

The dose of 40 mg/kg/day was obtained from the dose-response curve of our pilot study, which is equivalent to the human dose of 400 mg/day in an average adult. A low dose (10 mg/kg) was obtained as 25% of the actual dose.

### Sample collection and animal sacrifice

Twenty-four hours after the last treatment, the rats were euthanized with sodium pentobarbital (30 mg/kg) intraperitoneally. Blood samples were obtained by cardiac puncture and the testes and epididymis were excised.

### Sperm analysis

Sperm suspension was obtained from the left caudal epididymis using the diffusion method and analyzed using a microscope (Olympus, Japan) as described by Obembe *et al.* (2023). The sperm suspension obtained was diluted with 0.5 mL Tris buffer solution and an aliquot of this solution was examined on a microscope slide (400 x). Sperm count was determined using the newly improved Neubauer’s counting chamber (hemocytometer). The ruled part of the chamber was focused and the number of spermatozoa counted in five 16-square cells. Progressive sperm motility estimates were performed from three different fields in each sample and the mean of the three estimates was used as the final motility score. Sperm viability was assessed using the eosin/nigrosin staining technique. Viable sperm cells remained unstained, while the dead sperm cells were stained. Based on these observations, percentile viability was recorded. The sperm morphology was considered abnormal when an anomaly was observed on the tail, neck, or head and was expressed as a percentage of morphologically normal sperm. Epididymal volume was estimated by immersing the epididymis in 5 mL of normal saline in a measuring cylinder. The volume of fluid displaced was recorded as the epididymal volume (Seed *et al.*, 1996).

### Determination of oxidative stress markers of the serum and testis

The blood collected was centrifuged at 3,000 rpm for 5 minutes and the serum obtained was refrigerated. Also, the right testis was weighed and homogenized in phosphate buffer (pH 7.4). The homogenates were centrifuged at 10,000 rpm for 10 minutes in a cold centrifuge at 4^o^C. The supernatant was carefully decanted and preserved at -20^◦^C. Oxidative stress markers *viz.* malondialdehyde (MDA), superoxide dismutase (SOD), glutathione peroxidase (GPx), and catalase were assessed in the serum and testicular homogenate obtained. Briefly, MDA was determined using the method described by Stocks & Dormandy (1971). One ml of the sample was combined with 2 ml of TCA-TBA-HCL, mixed thoroughly, and heated for 15 minutes. After cooling, the fluorescent precipitate was removed by centrifugation. The absorbance of the sample was determined at 535 nm. SOD activity was determined by the method of Misra & Fridovich (1972). The samples were diluted with distilled water in a ratio of 1:9. An aliquot of 0.2 ml of the diluted sample was added to the 2.5 ml of 0.05M carbonate buffer. The increase in absorbance at 480 nm was monitored every 30 sec for 150 sec. GPx level was measured according to the method described by Hafeman *et al*. (1974). The sample was incubated with 5 mM GSH, 1.25 mM H_2_O_2_, 25 mM NaN_3_, and phosphate buffer. After the reaction was stopped, 2 ml of the supernatant was mixed with Na_2_HPO_4_ and DTNB. The absorbance of the yellow-colored complex was measured at 412 nm after incubation for 10 minutes. Catalase activities were determined by the method described by Sinha (1972), 0.1 ml of the sample was mixed with 1.0 ml of 0.01M phosphate buffer (pH 7.4), and incubated for 10 minutes. After the reaction was stopped, the sample was centrifuged and the supernatant was used to quantify the amount of H_2_O_2_ to calculate catalase activity at 570 nm.

### Determination of hormonal levels

From the serum obtained, gonadotropin-releasing hormone (GnRH), follicle-stimulating hormone (FSH), luteinizing hormone (LH), and testosterone were measured using their respective ELISA kits. The left testis was homogenized using chloroform-methanol buffer in a cold centrifuge at 4^o^C. The post mitochondria fraction was decanted, and preserved at -20 ^◦^C, and thereafter, testicular testosterone was assayed. GnRH ELISA kit (Nanjing Mornmed Medical Equipment Co., Ltd, China), was used to assay GnRH. The procedure involved the preparation of reagents, adding standards and samples in duplicate, and incubating with HRP conjugate for 60 mins at 37°C. After 5 washes chromogen solutions were added followed by the stop solution, and absorbance was recorded at 450nm. A respective ELISA kit (Bio Inteco, UK) was used to assay FSH, LH, and testosterone. This procedure involved setting up standard, pilot samples and control wells on the pre-coated plates, with a duplicate for each. After washing 50µl of standards, samples, and control were added followed by 50µl of biotin-labeled antibody solution. The plates were incubated at 37°C for 45 minutes, washed three times, and incubated with HRP- streptavidin conjugate for 30 minutes. After 5 washes 90 µl of TNB substrate was added and the plate was incubated in the dark for 10 to 20 minutes, the reaction was terminated with 50µl of stop solution, and the absorbance was read at 450nm using a microplate reader.

### Measurements of inflammatory enzymes and cytokines

Serum LDH, interleukin-1β (IL-1β), interleukin-6 (IL-6), interleukin-10 (IL-10), and tumor necrosis factor-α (TNF-α) were measured using their respective ELISA. LDH assay kit (Agappe Diagnostics, Switzerland) was used to determine the serum LDH activity, by mixing 10µl of the sample with 1000µl of working reagent, then incubated at 37°C for 1 min, the reaction was monitored by monitoring the change in absorbance per minute over a 3 min period. Interleukin-1β, 6, 10, and TNF-α ELISA kits (Nanjing Mornmed Medical Equipment Co., Ltd, China) were used to assay the cytokine levels in serum. The assay procedure involved setting up standards, samples, and control on a pre-coated plate, with a duplicate for accuracy. After 90 min incubation, 37°C and washing, biotin-labeled antibodies and HRP- streptavidin conjugate were added sequentially with washing steps. TNB substrate was then added, incubated for 10 to 20 mins, and stopped with 50µl stop solution. The absorbance was read at 450nm.

### Histological examination

After harvesting the testis tissues, it was fixed in a 10% neutral buffered formalin, it was later embedded in paraffin and 5 µm thick sections were prepared and stained with hematoxylin and eosin using standard procedures. The slides were viewed under a light microscope (Celestron LCD digital microscope, USA, model 44348) and photomicrographs were taken at 200× magnification.

### Statistical Analysis

All the values are expressed as mean±standard error of the mean (SEM). Data analysis was done using GraphPad Prism version 8.0.2 for Windows. The obtained data were subjected to D’Agostino Pearson Omnibus and Shapiro-Wilk’s test to test for normality distribution; hence, the differences between groups were analyzed by one-way ANOVA followed by Bonferroni *post-hoc* test. Differences were considered significant when *p*<0.05.

## RESULTS

### Effects of acyclovir on sperm parameters

Acyclovir (10 and 40 mg/kg) caused a significant decrease in sperm motility and sperm count in treated rats when compared with those of the control. However, the observed decline in sperm motility and sperm count was not dose-dependent. Also, the percentage of sperm cells with abnormal morphology significantly increased in acyclovir-treated rats when compared with the control in a dose-independent manner. However, the sperm viability and volume were not affected by acyclovir treatment (Table 1).

**Table 1:** Effects of acyclovir on sperm parameters.

	Motility (%)	Viability (%)	Volume (ml)	Sperm Count(×10^6^m/l)	Abnormal Morphology (%)
**Control**	83.57±3.22	95.14±1.52	5.16±0.02	124.86±2.69	11.54±0.38
**Acyclovir (10mg/kg)**	75.86±1.84^a^	94.86±1.74	5.17±0.02	116.29±2.98^a^	13.26±0.33^a^
**Acyclovir (40mg/kg)**	62.86±1.84a	95.29±1.80	5.17±0.02	95.43±5.11^a^	13.72 ±0.35^a^

Each value is an expression of mean ± SEM (*p*<0.05).

a - Values were significant when compared to the control group.

### Effects of acyclovir on oxidative stress markers, LDH, and inflammatory cytokines

Acyclovir therapy at 40 mg/kg led to significantly higher MDA levels and lower SOD and GPx activities in the serum when compared with the control but not in the 10 mg/kg acyclovir-treated rats. However, serum catalase was not significantly altered (Table 2). Also, acyclovir (40 mg/kg)-treated rats had significantly higher levels of MDA but reduced activities of SOD and catalase in the testes when compared with those of the control (Table 3). The apparent changes in these oxidative markers in the serum and testes of rats treated with the lower dose of acyclovir were not statistically significant.

**Table 2:** Serum oxidative stress markers of acyclovir treated rats.

	MDA (µM/g)	SOD (U/mg)	GPx (µ/ml)	CAT (U/mg)
**Control**	0.44±0.20	4.58±0.92	4.09±0.77	5.27±0.87
**Acyclovir (10mg/kg)**	1.08±0.22	3.16±0.44	2.71±0.85	3.86±0.75
**Acyclovir (40mg/kg)**	1.44±0.19^a^	1.82±0.30^a^	1.40±0.47^a^	2.6114±0.52

Each value is an expression of mean ± SEM. (*p*<0.05).

a - Values were significant when compared to the control group.

**Table 3:** Testicular oxidative stress markers of acyclovir treated rats.

	MDA (µM/g)	SOD (u/mg)	CAT (u/mg)
**Control**	1.01±0.06225	3.80±0.5148	7.319±1.174
**Acyclovir (10mg/kg)**	1.15±0.09025	2.74±0.6384	5.931±0.8285
**Acyclovir (40mg/kg)**	1.67±0.1738 ^a,b^	1.80±0.3000^a^	3.261 ±0.3865^a^

Each value is an expression of mean ± SEM. (*p*<0.05).

a - Values were significant when compared to the control group,

b -Values were significant when compared to 10mg/kg Acyclovir

Serum LDH, IL-6, and TNF-α were significantly higher in acyclovir (40mg/kg)-treated rats while IL-10 was significantly lower when compared with the control. IL-1β was not significantly altered following acyclovir treatment (Table 4).

**Table 4:** Inflammatory enzyme and cytokines of acyclovir treated rats.

	LDH (u/l)	IL-1β (pg/mL)	IL-6 (pg/mL)	IL10 (pg/mL)	TNF-α (pg/mL)
**Control**	24.24±2.78	18.04±1.71	52.90±11.61	18.44±1.91	228.80±15.21
**Acyclovir (10 mg/kg)**	37.77±4.30	19.72±0.81	77.90±14.64	14.72±1.7	247.30±18.43
**Acyclovir (40 mg/kg)**	58.90±6.65 ^a,b^	21.56±1.55	100.5±8.256^a^	12.26±0.74^a^	315.50±23.80^a^

Each value is an expression of mean ± SEM. (*p*<0.05).

a - Values were significant when compared to the control group,

b -Values were significant when compared to 10mg/kg Acyclovir.

### Effects of acyclovir on sex hormones

Serum levels of GnRH, FSH, LH, and testosterone and testicular concentration of testosterone were significantly reduced in rats that had acyclovir at 40mg/kg, but not at 10 mg/kg, when compared with the control animals (Table 5).

**Table 5:** Reproductive hormones of acyclovir treated rats.

	GnRH (mIU/mL)	FSH (mIU/mL)	LH (mIU/mL)	Serum testosterone (ng/mL)	Testicular testosterone (ng/mL)
**Control**	42.58±4.14	78.17±4.54	61.50±7.01	1.467±0.159	3.800±0.23
**Acyclovir (10mg/kg)**	33.75±1.44	66.50±5.15	56.33±3.62	0.8667±0.24	3.85±0.12
**Acyclovir (40mg/kg)**	30.92±1.45^a^	53.83±3.62^a^	40.17±5.17^a^	0.5833±0.189^a^	3.120±0.091^a,b^

Each value is an expression of mean ±SEM. (*p*<0.05).

a - Values were significant when compared to the control group,

b -Values were significant when compared to 10 mg/kg Acyclovir.

### Effects of acyclovir on testicular histoarchitecture

**Figure 1:**
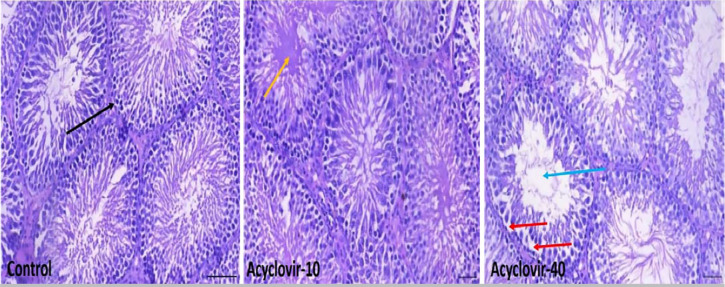
Testicular histoarchitecture of acyclovir treated rats (Magnification X 200, H/E staining, Scale Bar = 20µm). A. (Control) is normal testis showing numerous seminiferous tubules (black arrow) with germ cells at varying degree of maturation. B. (10 mg/kg acyclovir): there are edematous seminiferous tubules (yellow arrow). C. (40 mg/kg acyclovir): showed some seminiferous tubules with degenerated spermatogenic cells (red arrow), and scanty sperm cells in the lumen (blue arrow).

The rats in the control group had normal testicular tissue, that is composed of numerous seminiferous tubules with germ cells at varying maturation degrees. In the 10mg/kg acyclovir-treated group, the seminiferous tubules appeared edematous. In the 40mg/kg acyclovir-treated group, the seminiferous tubules showed degenerated spermatogenic cells and scanty sperm cells in the lumen (Figure 1).

## DISCUSSION

Previous studies on antiretroviral, including acyclovir, demonstrated the cytotoxic effect of these drugs (Narayana, 2008; Akhigbe *et al.*, 2021, 2024). Thust *et al.* (1996) also reported the clastogenic activity of acyclovir. Clastogenic substances cause DNA damage and chromosomal abnormalities; thereby affecting cell homeostasis and causing dysfunction or cell death (Bolzán, 2020). This damage can trigger an immune response and the immune system will respond to damaged or dying cells by activating inflammatory pathways to remove the affected cells and initiate tissue repair. DNA damage and chromosomal abnormalities can also activate cellular stress responses (Bakkenist & Kastan, 2004), including pathways that lead to the production of pro-inflammatory cytokines as seen by an increase in IL-6 and a decline in anti-inflammatory cytokines as seen by the reduction of IL-10 in this study. These pro-inflammatory molecules can recruit immune cells to the site of damage, resulting in inflammation (Schnoor *et al.*, 2016). Cells that experience significant chromosomal damage may enter a state known as cellular senescence. Senescent cells often secrete a variety of pro-inflammatory cytokines, chemokines, and proteases, collectively referred to as the senescence-associated secretory phenotype. This can contribute to a local inflammatory environment and cause the release of reactive oxygen and nitrogen species (dAddadiFagagna, 2008; Sikora *et al.*, 2021), leading to oxidative stress and depletion of antioxidant enzymes as observed in the serum and testes of acyclovir-treated rats in this study.

The relationship between inflammatory mechanisms and oxidative stress is complex and correlational. These occurrences mutually enhance one another, resulting in the formation of a positive feedback loop (Lugrin *et al.*, 2014; Patergnani *et al.*, 2021; Akhigbe *et al*. 2024). Likely, acyclovir-induced inflammation via the upregulation of pro-inflammatory cytokines (TNF-α, IL-1β, and IL-6) and downregulation of anti-inflammatory cytokine (IL-10) activated the generation of considerable amounts of reactive oxygen and nitrogen species, which caused the depletion of antioxidant enzyme levels and activities and increased malondialdehyde release, leading to oxidative stress. On the flip side, oxidative stress can damage cellular components, provoking the release of pro-inflammatory mediators and the activation of transcription factors that regulate the expression of pro-inflammatory genes (Biswas, 2016; Soomro, 2019; Mahmoud *et al.*, 2021). Hence, acyclovir may induce increased reactive oxygen and nitrogen species generation, leading to the upregulation of pro-inflammatory cytokines.

The observed increase in testicular oxidative stress seen in the treated rats may also be due to the inhibition of DNA synthesis by acyclovir. This inhibition possibly led to the disruption of the cellular homeostatic system, which causes alteration of metabolic pathways, leading to the development of oxidative stress. Also, the inhibition of DNA synthesis may cause mitochondrial dysfunction which is a major player in the development of oxidative stress, through the increase in the production of reactive oxygen species (ROS) (Abid-Essefi *et al.*, 2004; Martins *et al.*, 2021). The increase in MDA affirms an increase in lipid peroxidation and consequently the depletion of antioxidant enzymes (Adnan *et al.*, 2019). The depletion of testicular antioxidants promotes ROS attack, leading to distorted testicular histomorphology.

The cellular damage caused by clastogenic agents sets off a cascade of events that activate and sustain inflammatory responses, ultimately contributing to various inflammatory diseases (Chen *et al.*, 2018). Lactate dehydrogenase is an enzyme involved in energy production by conversion of lactate to pyruvate and it is present in almost all body cells with the highest levels in the heart, liver, lungs, muscles, kidneys, and blood cells. It is a general indicator of acute or chronic tissue damage and it is considered an inflammatory marker (Barrak *et al.*, 2024). In the testis, it drives the production of ATP that is required to maintain optimal spermatogenesis and keep the germ cells viable. The observed acyclovir-induced rise in the level of LDH correlates with reports from Jagetia *et al.* (2000); and may be linked with the altered testicular histoarchitecture and degeneration of germ cells. Inflammatory conditions activate immune cells such as macrophages and neutrophils.

Acyclovir was observed to significantly decrease testosterone levels both in the serum and in the testis. The decline in testosterone levels shows that acyclovir has anti-androgenic properties. One of the major mechanisms of action of acyclovir is the inhibition of DNA synthesis, which may cause the blockage of androgen action in the cells of the male reproductive system (Semet *et al.*, 2017). Acyclovir may indirectly reduce DNA synthesis in these cells by halting androgen-dependent cell proliferation, causing cell cycle arrest, which culminates in the reduction of DNA synthesis, thereby inducing apoptosis in androgen-dependent cells, altering metabolic pathways. This can indirectly affect the cell’s capacity for DNA synthesis, leading to both a defect in spermatogenesis and steroidogenesis, as seen in this study. The decline in spermatogenesis is eminent, and expressed as a decline in sperm count and reduction of spermatogenic cells in addition to seminiferous tubules, with an almost empty lumen in acyclovir-treated rats.

Although acyclovir might induce direct testicular damage, the associated decline in GnRH, FSH, and LH shows that acyclovir impairs the hypothalamic-pituitary-testicular axis. Neurons are particularly sensitive to energy deficits due to their high metabolic demands and the depletion of antioxidant enzymes in the neurons will lead to an increase in ROS, causing energy depletion (as seen in the level of antioxidant enzymes in the serum) (Beckhauser *et al.*, 2016). Since acyclovir interferes with neuronal mitochondrial function (Brandariz-Nuñez *et al.*, 2021) it may inhibit mitochondrial DNA polymerase gamma, which is crucial for mitochondrial DNA replication and repair, leading to reduced ATP production, energy depletion, and cellular oxidative stress (DeBalsi *et al.*, 2017). This may alter GnRH neuronal function causing interference with the synthesis and release of GnRH from the hypothalamus (Wierman *et al.*, 2011), which will in turn affect the production and release of LH and FSH, and culminate in reduced testicular and circulating testosterone (Casteel & Singh, 2023).

Despite the convincing data presented in this study, it has some limitations. First, this is an experimental study using a Wistar rat model, which limits the extrapolation of these findings to humans, because of possible differences in drug metabolism and reproductive function between species. Also, acyclovir administration was for 21 days in the present study. Thus, the findings presented here may not reflect the long-term effects in humans who are on this medication for extended periods. More so, the controlled laboratory environment may not adequately account for other environmental factors that may influence male reproductive health in a real-world setting.

In conclusion, acyclovir (at 40mg/kg bw/day) induces testicular damage by promoting an inflammatory response, oxidative damage, and depletion of antioxidant enzymes. This was associated with impaired hypothalamic-pituitary-gonadal axis. This may pose a challenge to male patients on this drug regarding fertility and reproductive health. Clinical studies validating these findings and evaluating adjuvant therapies that may ameliorate the reproductive health consequences of acyclovir are recommended.
